# Temporal Processing of Audiovisual Stimuli Is Enhanced in Musicians: Evidence from Magnetoencephalography (MEG)

**DOI:** 10.1371/journal.pone.0090686

**Published:** 2014-03-03

**Authors:** Yao Lu, Evangelos Paraskevopoulos, Sibylle C. Herholz, Anja Kuchenbuch, Christo Pantev

**Affiliations:** 1 Institute for Biomagnetism and Biosignalanalysis, University of Münster, Münster, Germany; 2 German Center for Neurodegenerative Diseases, Bonn, Germany; University of California, Merced, United States of America

## Abstract

Numerous studies have demonstrated that the structural and functional differences between professional musicians and non-musicians are not only found within a single modality, but also with regard to multisensory integration. In this study we have combined psychophysical with neurophysiological measurements investigating the processing of non-musical, synchronous or various levels of asynchronous audiovisual events. We hypothesize that long-term multisensory experience alters temporal audiovisual processing already at a non-musical stage. Behaviorally, musicians scored significantly better than non-musicians in judging whether the auditory and visual stimuli were synchronous or asynchronous. At the neural level, the statistical analysis for the audiovisual asynchronous response revealed three clusters of activations including the ACC and the SFG and two bilaterally located activations in IFG and STG in both groups. Musicians, in comparison to the non-musicians, responded to synchronous audiovisual events with enhanced neuronal activity in a broad left posterior temporal region that covers the STG, the insula and the Postcentral Gyrus. Musicians also showed significantly greater activation in the left Cerebellum, when confronted with an audiovisual asynchrony. Taken together, our MEG results form a strong indication that long-term musical training alters the basic audiovisual temporal processing already in an early stage (direct after the auditory N1 wave), while the psychophysical results indicate that musical training may also provide behavioral benefits in the accuracy of the estimates regarding the timing of audiovisual events.

## Introduction

Multisensory events, such as watching and listening to an opera or a concert, are mostly perceptually integrated and recognized as having synchronous audiovisual information even when perceived from a distance. Nevertheless sound travels much slower than light in the air, and therefore the visual and auditory information of a distant event are actually asynchronous. This tolerance in recognizing the timing differences of multisensory events helps us to avoid focusing unnecessary attention to this phenomenon in daily perception. Asynchronies greater than this tolerance window, such as perceptive differences between seeing a lightning and hearing the corresponding thunder, are mostly recognized as two different events.

Pitch and rhythm are two primary components of music. Appreciation of music is partly based on generating rhythmic expectations and processing the multiple temporally coordinated auditory events. Compared to merely listening to music, practicing a musical instrument requires complex multisensory processing involving simultaneous integration and interaction of visual, auditory, somatosensory and motor information [1], [2]. In order to master precise rhythm and tempo variations, musicians often use a metronome to pace their actions when practicing. Orchestral musicians rely more on advanced multimodal skills. They not only have to coordinate and integrate their motor actions with visual, auditory and proprioceptive feedback from their own instrument and from the musical score, but they have also to attend to and synchronize their actions with those of their fellow musicians (using visual and auditory information) and with the conductor's gestures (visual) as well. Apart from pitch and dynamics, precise timing is among the greatest challenges in orchestral music making. Numerous studies have demonstrated structural [3], [4] and functional [1], [3], [5]–[9] differences between professional musicians and non-musicians in brain areas related both to specific sensory and to multisensory integration domains. [1], [2], [9]–[11]. The musicians benefit from their long term musical training at multiple levels of cortical processing. Particularly, in comparison to non-musicians, they have pronounced auditory cortical representations for tones of the musical scale [12]–[16], superior ability for musical imagery [17], enhanced cortical representation for musical timbre [18] and increased sensorimotor responses [19], [20].

Since musical performance requires precise processing of temporally correlated multisensory events, musicians' long term training can reveal novel insights regarding temporal binding of multiple senses. Multiple psychophysical investigations demonstrate that long term musical training improves temporal binding of auditory and visual information. For example, Jazz drummers have advanced ability to detect audiovisual asynchrony, especially for slower drumming rhythms [21]. Electrophysiological reports also showed enhanced temporal and frequency encoding of audiovisual information in the brainstem of musicians viewing videos of speech and music [22]. In a combined psychophysics–fMRI study comparing controls and musicians [23], the later showed a narrower temporal integration window as measured behaviorally along with increased audiovisual asynchrony BOLD responses. This was the case selectively in a musical, but not a linguistic task, which indicated that long term musical training alters precise estimates of the temporal audiovisual timings specifically for music.

A large body of fMRI studies found that audiovisual (a)synchrony processing relies on a widespread neural network mainly including subcortical, primary sensory, cerebellar, and premotor areas [23]–[26]. Nevertheless, little is known about the way how long term musical practice alters temporal processing of audiovisual information. Using the advantage of precise temporal resolution of the MEG we were able to investigate the temporal integration and interaction of auditory and visual stimuli at a narrow time window of 50 ms and at relatively early stage of brain processing (direct after the auditory N1 response). Professional musicians were recruited for this combined psychophysical and neurophysiological study in order to investigate the initial stage of multimodal temporal processing with a hypothesis that their long-term multisensory experience alters temporal audiovisual processing already at an early stage. For this purpose, a paradigm was used that was composed from non-musical audiovisual events presented either synchronously or in various levels of asynchrony. Thereby we intended to investigate the neural correlates of temporal processing of audiovisual information, and how the behavioral and neural correlates of temporal integration of audiovisual events are shaped by experience.

## Methods

### Ethics Statement

All subjects were fully informed about the execution and the goal of the study and gave written informed consent in accordance with procedures approved by the Ethics Committee of the Medical Faculty of the University of Münster (Ethics approval 5V Pantev (A)). This has been documented for each person individually. The study was performed in accordance with the Declaration of Helsinki.

### Subjects

Twenty-nine healthy subjects (15 musicians and 14 non-musicians) participated in the present study. The musicians were students of the Music Conservatory of Münster who had received instrumental lessons for a minimum of 12 years and were actively playing their instrument at the time of study. Non-musicians were students of various faculties of the University of Münster and were selected based on the fact that they never received musical education apart from the compulsory music lessons in school. All participants were right handed according to the Edinburgh Handedness Inventory [27] and had normal hearing as tested by clinical audiometry. Four subjects were excluded from the data analysis. Two of them (one musician and one non-musician) were excluded due to excessive head movement during the MEG measurement. The other two (musicians) were excluded because they failed the control task included in the behavioral test (see the Design section). Thus, twelve musicians (eight female, four male; aged 19–29; mean ±SD: 22.25±3.08 years) and thirteen non-musicians (nine female, four male; aged 23–31; mean ±SD: 26.15±2.85 years) were included in the final data analysis.

### Design

**Figure 1:**
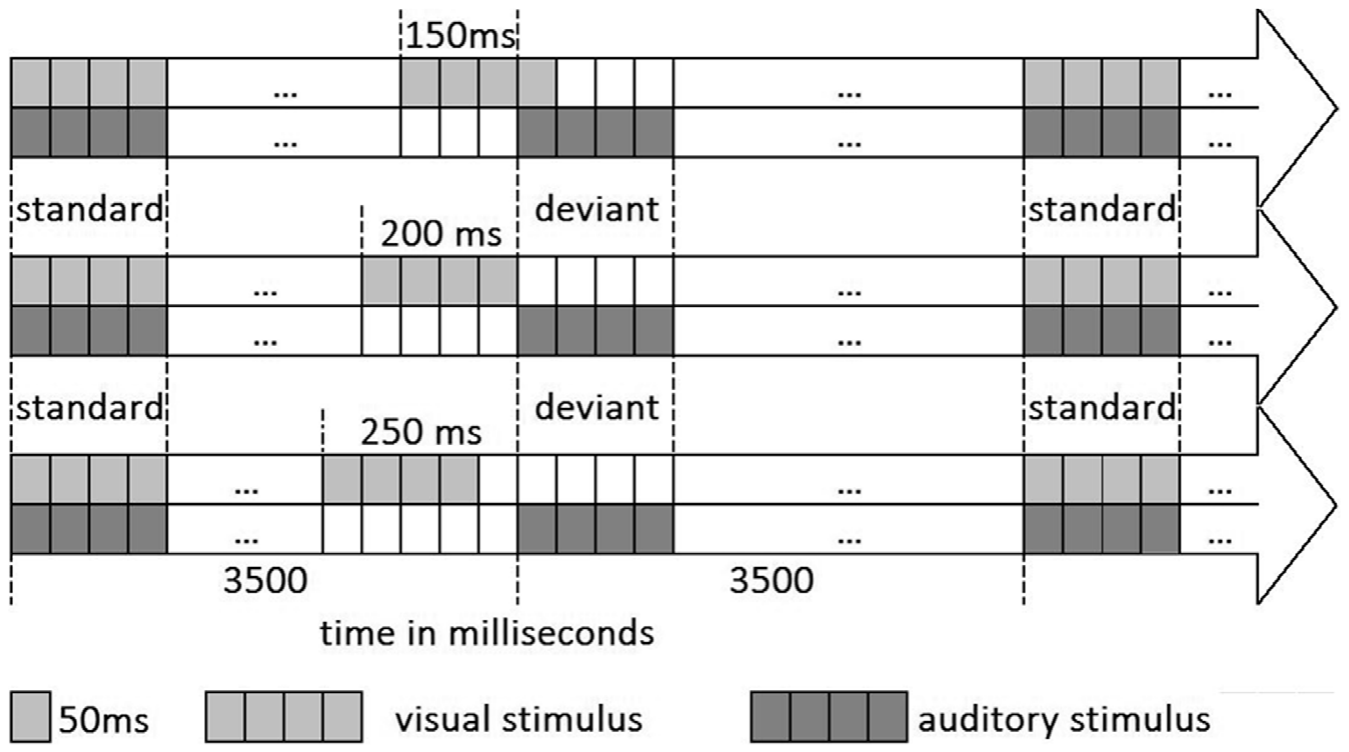
Illustration of the design. Each row represents one run. The auditory stimulus was presented with a stable SOA while the visual stimulus with a varying one creating asynchronous audiovisual stimuli of 150

Synchronous and asynchronous audio-visual stimuli were used for the behavioral and neurophysiological MEG testing. The auditory part of all stimuli consisted of a sinusoidal tone of 880 Hz (duration of 200 ms including 10 ms rise and decay time). The interstimulus interval between subsequent tones was always 3500 ms (c.f. figure 1). A black circular dot (RGB: 255, 255, 255) positioned in the middle of a continuously presented gray background (RGB: 125, 125, 125) presented with the same duration of 200 ms as the tone was used for the visual part of the stimuli (c.f. figure 1). The simplicity of the stimulation was chosen because it does not favor prior musical experience, as it would be the case for visible finger movements and concurrent piano tones [23]. In order to assess the subject's compliance to the task (see the behavioral measurements section) a control condition was included. In this control condition the auditory and the visual part were presented simultaneously, but the visual part was altered by having more smoothed, indistinct edge compared to the visual part of the stimuli in the experimental conditions. Participants who made more than 4 mistakes in the control condition within one run (5 of total 10 trials, i.e. 50%) were excluded from the data analysis.

The simultaneously presented auditory and visual stimuli formed the synchronous condition. In the asynchronous conditions we used three different levels of difficulty with asynchronies of 150 ms, 200 ms, or 250 ms with the visual part of the stimulus preceding the auditory one. The three different asynchrony levels (c.f. figure 1) were performed in three runs, respectively, with short breaks in between. They were presented in a pseudo-randomized order to each subject, balanced across the two groups of musicians and non-musicians. Ten trials of the control condition were randomly presented during each run. Each run consisted thus, of 140 synchronous, 69 asynchronous and 10 control trails. The duration of each run was 13 minutes and the complete experiment lasted 45 minutes.

### Behavioral measurements

Behavioral measurements were integrated into the MEG measurements. After the presentation of each trial, subjects had to judge if it was a synchronous, an asynchronous or a control trial and indicated their decision by pressing one of the three corresponding mouse buttons. The test subjects were instructed to judge the trails as accurate as possible and were requested to react after a pause of 1.5 to 2 seconds in order to avoid interference of the muscle activity with the MEG activity. Correct and incorrect responses from all conditions were averaged respectively for each run. This was done to investigate whether musicians have an advantage when performing the harder task. The responses were also averaged across conditions for all three runs in order to test whether musicians are better than non-musicians in total, independently from the levels of asynchrony. The missed button presses were regarded as incorrect responses. The results of the control condition were used merely to judge the subject's compliance to the task (see the Subjects section). Therefore, they are excluded from the final behavior and MEG analysis. The results of the two other conditions (synchrony and asynchrony) were used for the following statistical analysis of the behavioral data.

### MEG recordings

Evoked magnetic responses were recorded using a 275 channel whole-head system with inter-channel spacing of 2.2 cm (OMEGA, CTF Systems Inc., Port Coquitlam, Canada) in an acoustically silent and magnetically shielded room. Participants were comfortably seated upright and their head position was stabilized with cotton pads. MEG data were obtained continuously during each presentation run, low-pass filtered at 150 Hz and sampled at a rate of 600 Hz.

The auditory stimuli were delivered via air conduction through two plastic tubes of 90 cm length at intensity of 60 dB above the individual hearing threshold, which was individually determined for each ear at the beginning of each MEG session with an accuracy of 5 dB. The visual stimuli were projected onto the back of a semi-transparent screen positioned 90 cm in front of the subjects' nasion with an Optoma EP783S DLP projector and a refresh rate of 60 Hz. During the session the, subjects were continuously monitored. In order to minimize artifacts, subjects were instructed to keep still and try to blink and swallow if necessary between trials. Subjects were also instructed to keep their eyes open and fixate on the middle of the screen.

### Data analysis

The Brain Electrical Source Analysis software (BESA Research, version 5.3.7; Megis Software) was used for preprocessing and source analysis of the MEG data. The continuous MEG recordings were divided into epochs of 900 ms, starting 400 ms before and ending 500 ms after the tone onset. Data were filtered with a high-pass filter of 1 Hz, a low-pass filter of 30 Hz, and additional notch filter at 50 Hz. Epochs were baseline-corrected using the interval from −350 to −250 ms before the tone onset. The baseline interval was choses so in order not to include the preceding visual stimulus in any of the asynchronous conditions. Epochs containing signals larger than 2.5 pT were considered artifact-contaminated and excluded from the averaging. Averages of all three runs were computed separately for each subject for the audiovisual synchronous and asynchronous conditions. Control stimuli were not included in the MEG data analysis. Only the synchronous trials before the asynchronous ones were included in the final analysis.

**Figure 2:**
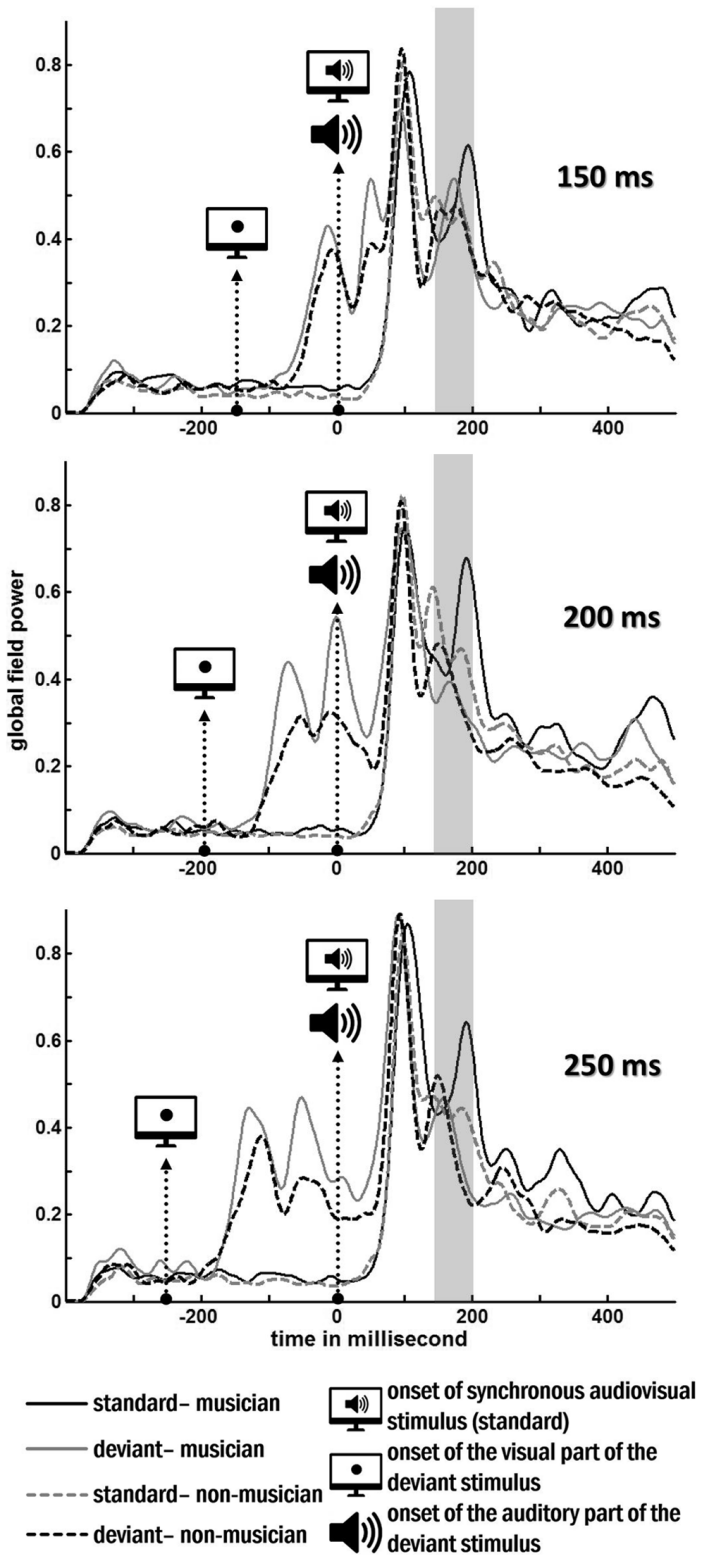
Grand averaged global field power for the responses musicians (continuous lines) and non-musicians (dashed lines) for synchronous and asynchronous stimuli. The gray bar indicates the time interval where the analysis was performed.

In order to localize the sources of the neural responses of each subject and each stimulus category (audiovisual synchrony, audiovisual asynchrony, musicians, non-musicians), the low-resolution brain electromagnetic tomography (LORETA) [28] method was used. LORETA calculates distributed Current Density Reconstructions (CDR) throughout the full-brain volume. This method has the advantage of not needing an a priori assumption of the number of activated sources. The appropriate time window for the CDR was chosen to include the time window that showed most overall activity after the N1 as seen in the grand averaged global field power. This definition led to a time window of 50 ms (c.f. figure 2, 150 ms–200 ms after the tone onset) and was common for all conditions. This time window is typically chosen for audiovisual mismatch responses and it is within the range of the audiovisual MMN latency [29], [30]. Using BESA we calculated the mean CDR image of the selected time window for each individual and each condition. The images were then projected onto a standard MRI template, based on the Montreal Neurological Institute (MNI) template. Images were smoothed and their intensities normalized by convolving an isotropic Gaussian kernel filter with 7 mm full width half-maximum.

Statistical Parametric Mapping 8 (SPM8, http://www.fil.ion.ucl.ac.uk/spm) and GLM Flex (http://nmr.mgh.harvard.edu/harvardagingbrain/People/AaronSchultz/GLM_Flex.html) analysis packages were used for the statistical analysis of the CDRs. Using GLM Flex, a 2×2×3 flexible factorial model was designed to explore the main effects of group, condition, and latency and the group × condition × latency interaction. The flexible factorial model is GLM Flex equivalent analysis to a mixed-model 3-way ANOVA comparison. The factors included in the analysis were group (musicians and non-musicians), condition (synchrony and asynchrony) and latency (150 ms, 200 ms and 250 ms).

Results were masked using a gray matter mask in order to keep the search volume small and in physiologically reasonable areas. In order to control the multiple comparisons, we used a permutation method for peak-cluster level error correction (AlphaSim) at 5% level, as implemented in REST software [31], by taking into account the significance of the peak voxel (threshold, p<0.001 uncorrected) along with the cluster size (threshold size >178 voxels). The smoothness parameter entered in AlphaSim was calculated from the residual image of the 3 way ANOVA.

## Results

### Behavioral results

**Figure 3:**
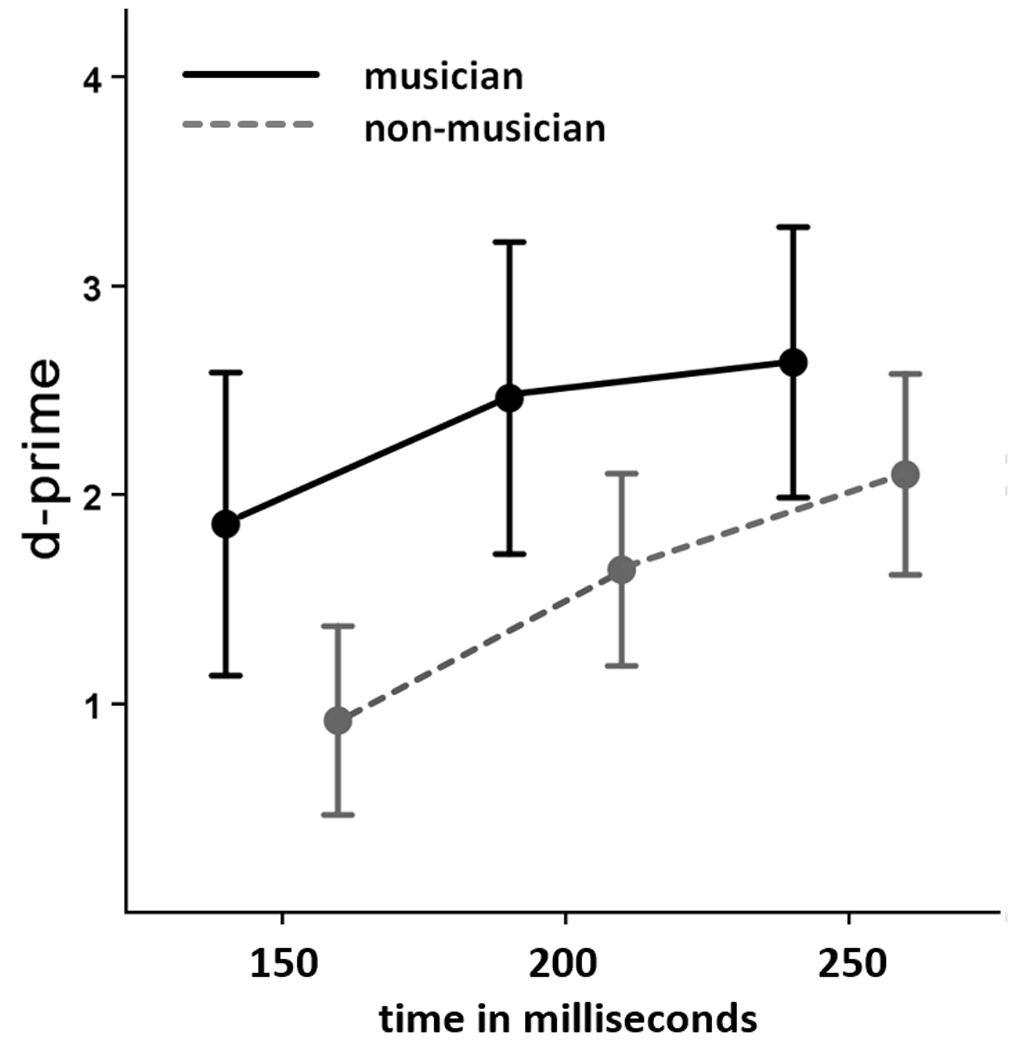
Behavioral results indicating discriminability of the three different latency conditions for musicians (continuous black line) and non-musicians (dashed gray line). Error bars show 95% confidence interval.

The discriminability index, *d* -prime, was used to evaluate the behavioral responses. The 2×3 way mixed-model ANOVA with between-subject factor group (musicians and non-musicians) and within subject factor asynchrony (150 ms, 200 ms, 250 ms) revealed a main effect of group [*F* (1, 23) = 4.643; *p* = 0.042] and a main effect of time differences (*F* (2, 46) = 36.555; *p* = 0.001). The interaction revealed no significant effects. In order to define the direction of the group effect, an independent samples t-test was calculated, post hoc, revealing that musicians identified the synchronous and asynchronous stimuli significantly better than non-musicians [*t* (23) = 2.155; *p* = 0.042]. Similarly, in order to identify the direction of the main effect of the time differences, paired samples t-tests were calculated, post hoc, comparing the three different time differences. The comparison of 150 ms with 200 ms indicated that the 200 ms condition was more reliably identified as asynchronous than the 150 ms condition [*t* (24) = −5.933; *p* = 0.001]. Additionally, the 250 ms condition was more easily identified as asynchronous than the 200 ms one [*t* (24) = −3.141; *p* = 0.004], indicating that independently of the group the bigger the time difference between the visual and auditory stimulation, the more reliable the identification of the asynchrony (c. f. figure 3).

### MEG results

#### Condition comparison

**Figure 4:**
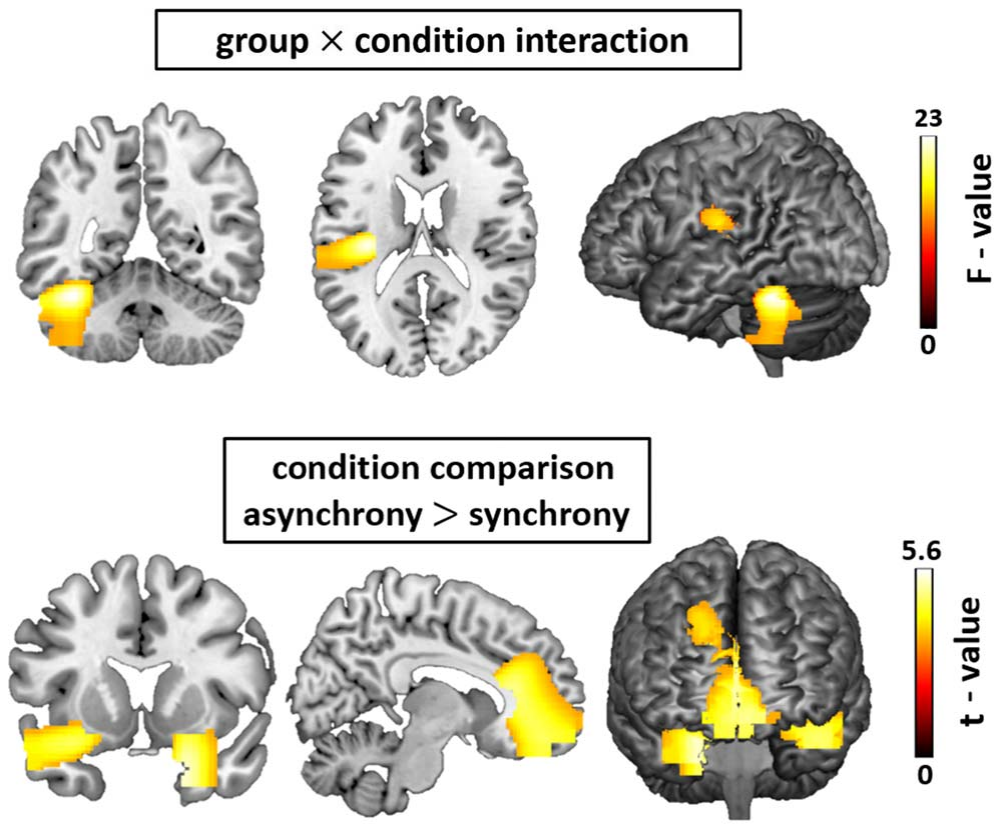
Statistical parametric maps of the musicians to non-musicians comparison and the audiovisual asynchrony response, as revealed by the flexible factorial model. Threshold: AlphaSim corrected at p<0.05 by taking into account peak voxel significance (threshold p<0.001 uncorrected) and cluster size (threshold size >178 voxels).

The main effect of condition was analyzed using a t-contrast because our intention was to identify the regions that had greater activity in the asynchronous conditions. The statistical analysis for this audiovisual asynchronous response revealed three clusters of activity. Specifically, the biggest cluster (size  = 5235 voxels) of activity was located in the in the Anterior Cingulate Cortex (ACC; peak coordinates: x = −1, y = 44, z = −5; *t* (23) = 5.09; p<0.05 AlphaSim corrected) extending to the Superior Frontal Gyrus (SFG). Two other clusters were located bilaterally in temporal regions. Activities on the right side were located in a relatively deep temporal region (peak coordinates: x = 18, y = −6, z = −12; *t* (23) = 5.54; cluster size  = 2014 voxels; p<0.05 AlphaSim corrected) extending to the right Superior Temporal Gyrus (STG) and Inferior Frontal Gyrus (IFG). Activities on the left side were located on the left STG (peak coordinates: x = −44, y = 22, z = −26; cluster size  = 1433 voxels; *t* (23) = 4.77; p<0.05 AlphaSim corrected) and IFG. The statistical map of these results is displayed in figure 4. The contrast showing greater activity in the synchronous condition than the asynchronous ones revealed three clusters of activity: The first cluster was located in the Cingulate cortex (size  = 1376 voxels; peak coordinates: x = −2, y = −24, z = 40; t (23) = 4.21; p<0.05 AlphaSim corrected) covering also a region in the inferior parietal cortex. Another cluster was located in the Right Cerebellum (size  = 3472 voxels; peak coordinates: x = 44, y = −70, z = −44; t (23) = 5.47; p<0.05 AlphaSim corrected) and the last one (size  = 9340 voxels; peak coordinates: x = 0, y = −94, z = −2; t (23) = 5.46; p<0.05 AlphaSim corrected) was covering the Lingual Gyrus and the Left Cerebellum.

#### Group × condition interaction

The 3-way interaction of group × condition × time differences revealed no significant activation difference indicating that the group effect did not differ according to the degree of asynchrony. Subsequently the 2×2 group × condition interaction was calculated in order to identify differences in the audiovisual asynchrony response, independently of the degree of asynchrony. For the statistical analysis of the group × condition interaction we used an *F*-contrast that revealed significantly different activity in two clusters located both in the left cortex. Specifically, one cluster (size  = 1868 voxels) was located in the left Cerebellum (peak coordinates: x = −49, y = −59, z = −25; F (2, 46) = 22.67; p<0.05 AlphaSim corrected) and the other one (size  = 822 voxels) was covering the left STG including the auditory cortex, the Postcentral Gyrus and the Insula (peak coordinates: x = −33, y = −19, z = 16; F (2, 46) = 22.92; p<0.05 AlphaSim corrected). The corresponding statistical map of this analysis is presented in figure 4. Subsequently, four separate t-contrasts were then calculated in order to show the direction of the differences found in the group × condition interaction. The t-contrast revealed that the cluster of activity difference located in the left STG originated from an enhanced activity of this region in the group of musicians when confronted to synchronous stimuli (peak coordinates: x = −34, y = −20, z = 16; *t* (25) = 4.83; p<0.05 AlphaSim corrected). On the contrary, the activity located in the left cerebellum originated from an increased activity of this region in the group of musicians when confronted to asynchronous stimuli (peak coordinates: x = −40, y = −52, z = −24; *t* (25) = 4.84; p<0.05 AlphaSim corrected). The calculated contrasts of non-musicians did not reveal significant activations.

## Discussion

Musical training relies strongly on audio–visual integration, particularly when reading musical notation and playing in a musical ensemble. Numerous studies have demonstrated that the structural [3], [4] and functional [1], [3], [5]–[9] differences between professional musicians and non-musicians are not only found within a single modality, but also with regard to multisensory integration [1], [2], [8]–[11]. Professional musicians are thus an ideal model for investigating the neurophysiological correlates of the temporal binding of auditory and visual information with regard to the hypothesis that long-term multisensory practicing alters temporal audiovisual representations.

The design of the experiment as demonstrated in figure 1 combined synchronous and asynchronous audiovisual stimuli in order to investigate the temporal audiovisual processing. For this propose, the auditory part of the stimuli was identical, in pitch and time, for all conditions. This stability ensured that the paradigm will not generate an auditory mismatch negativity response based on the auditory stimulus alone and therefore there will not be an interference with the temporal audiovisual asynchrony response [32], [33]. The only variable element is the timing of the appearance of the visual part of the stimuli, which is synchronous to the auditory part in one condition, while it is preceding the auditory part by 150 ms, 200 ms and 250 ms in the asynchronous conditions. Therefore, this paradigm was suitable for eliciting a differential response purely based on the audiovisual timing difference.

Behaviorally, musicians scored significantly better than non-musicians in judging whether the auditory and visual stimuli were synchronous or asynchronous, for all three latencies. This effect has been previously demonstrated in a more musical task using Jazz drummers that show advanced ability to detect audiovisual asynchrony[21]. Even short term perceptual temporal audiovisual training has been shown to narrow the size of multisensory temporal binding windows [34]. Alongside, this effect is present in other studies that investigate long term musical training effects in audiovisual temporal processing [35] and the musical task (but not the corresponding linguistic task) [23]. Interestingly our results show a non-significant increase of the difference between musicians and non-musicians as the time differences get smaller, allowing the hypothesis that if an even shorter latency difference was introduced an interaction would arise.

At the neural level, the statistical analysis for the audiovisual asynchronous response revealed three clusters of activations, generated in frontal and temporal regions. The activity evoked by the audiovisual asynchronous condition was greater than the one evoked by the synchronous one in a large cluster including the ACC and the SFG and two bilaterally located activations in IFG and STG.

Activations related with temporal audiovisual processing in these areas have been shown in several studies using a variety of neuroimaging techniques such as PET, fMRI and MEG. For example, the activation differences in IFG as seen in our study could be partly linked to a PET study [24] aiming to detect the cross-modal temporal integration of non-speech auditory and visual stimuli. In this study, bilateral IFG activation differences were found together with right inferior parietal, right Insula and left Cerebellum when the visual stimulus preceded the sound. In a cross-sectional fMRI study [36] with expert drummers and novices it was shown that expertise reduces brain activity for audiovisual matching actions. Using synchronized or desynchronized drumming strikes they found that the drummers' cortical activation was reduced in motor and action representation regions (i.e. bilaterally in the cerebellum and in the left temporal cortex) when the auditory and visual information was synced.

The ACC as well as SFG has been shown to have a functional relationship to attention [37], [38], expectancy deviation [39], various error detection tasks [40], conflict [41] and audiovisual integration [42], [43]. In our study, the audiovisual asynchrony occurred within the context of a paradigm that required attention, error detection and decision-making. These processes could be related to our finding of frontal activation differences. Activation differences in ACC, SFG and IFG in response to abstract audiovisual incongruities have also been recently shown using MEG [14]. Moreover in an fMRI study investigating the neural correlates of temporal audio-visual integration [43] activation differences were seen in the superior temporal sulcus and in the IFG.

An additional result of the neural asynchrony network as revealed by the comparison of the asynchronous to the synchronous stimuli of the present study was that musicians, most likely via their long-term musical practice, modified their basic neural processing of temporal audiovisual integration. The group differences in the MEG data are consistent with the behavioral benefit that musicians reveal. Taken together, they indicate an effect of long-term training on audiovisual processing. The areas that were found to have increased neuronal activity in the group of musicians were located in posterior temporal and cerebellar regions. These regions are known to be structurally and functionally affected by musical training [4], [23], [44], [45].

Musicians, in comparison to non-musicians, respond to synchronous audiovisual events with enhanced neuronal activity in a broad left posterior temporal region that covers the left STG, the Insula and the Postcentral Gyrus. Multiple studies investigating the neural basis of multisensory temporal processing identified a coherent network of areas that include the insula, the posterior parietal, and superior temporal cortices as being involved in the perception of audiovisual synchronicity [5], [24], [25], [43], [46], [47]. Further neurophysiological evidence [48], [49] demonstrates that these regions respond to multi-modal as compared to uni-modal stimuli with enhanced activation and also in their behavior the subjects are more accurate and rapid at identifying multimodal when compared with uni-modal objects [48], [49]. This network has been also found to be more responsive in musicians compared to controls in several studies [45], [50], and therefore it seems reasonable that neuroplastic changes in this region due to musical training affect the basic temporal multimodal processing.

Musicians showed significantly greater activity in the left Cerebellum when confronted with an audiovisual asynchrony. There are studies indicating that cerebellum has a central role in the control of perceptual and motor timing [51]–[55]. Penhune et al. [56] found that the function of the cerebellum in timing is conceptualized not as a clock or counter, but simply as the structure that provides the necessary circuitry for the sensory system to extract temporal information. For the motor system the cerebellum is important in order to learn to produce a precisely timed response. Alongside, the cerebellar volume has been found to have a positive correlation with long term intensity of musical practice [4] and it has been found to be significantly larger in musicians than in non-musicians [44].

In a recent MEG study of musicians and non-musicians [14] we have investigated the effect of incongruency based on the pitch height and it's graphic representation. Musicians showed greater activity in the right STG, a region contralateral to the region we have observed in our data. Several studies indicate that frequency and contour processing [3], [8], [57], [58] mainly involves the right auditory cortex, while rhythm [59] and time [60] elements are processed mainly in the left auditory cortex. Therefore the left lateralized activation evoked by the temporal characteristics in the present study seems reasonable. A similar fMRI study [23] investigated the temporal integration window of audiovisual synchrony specifically for speech and music. Partly in line with our results, the group comparison between musicians and non-musicians in the musical condition of this study indicated that musicians exhibited greater activation differences when confronted to audiovisual asynchrony in the left cerebellum, the left Superior Precentral Sulcus and the right posterior STG. In this study Lee and Noppeney investigated two highly modular systems (i.e. music and language) with specific characteristics while our study provides new results for a more basic level of temporal audiovisual processing. These differences, along with the inherent spatial and temporal differences of fMRI and MEG, may account for the opposite lateralization of the temporal activation.

Taken together, our MEG results form a strong indication that long-term musical training alters the basic audiovisual temporal processing already in an early stage (direct after the auditory N1 wave), while the psychophysical results indicate that musical training may also provide behavioral benefits in the accuracy of the estimates regarding the timing of audiovisual events.
